# Infratemporal abscess: a rare complication of a common procedure

**DOI:** 10.1099/acmi.0.000721.v3

**Published:** 2024-02-16

**Authors:** Rajesh Kotagiri, Matthew L. Repp, Michael H. Kim, Faissal Stipho

**Affiliations:** ^1^​ Banner University Medicine, Tucson, AZ, 85724, USA; ^2^​ Internal Medicine, University of Arizona, Tucson, AZ, 85724, USA; ^3^​ College of Medicine, University of Arizona, Tucson, AZ, 85724, USA

**Keywords:** coaggregation, *Eikenella corrodens*, Infratemporal abscess, *Streptococcus anginosus*, wisdom teeth extraction

## Abstract

The infratemporal fossa (ITF) is an anatomically complex cavity that houses a variety of muscular and neurovascular structures at the base of the skull. Infections involving the ITF, though uncommon, can be fatal due to the difficulties of accessing this anatomical space and its proclivity to evolve into a cavernous venous thrombosis (CVT). As a result, a multi-disciplinary approach involving several surgical and medical subspecialists is often warranted. We present a case of an infratemporal fossa abscess (IFA) after wisdom teeth extraction with a very complicated clinical course and a distinct microbiologic profile.

## Data Summary

No new data tools, software, or code has been generated or is required for work to be reproduced.

## Introduction

The infratemporal fossa is posterior to the maxillary sinus, medial to the zygomatic arch, lateral to the pterygoid plate, and anterior to the mastoid process [1]. This anatomically complex cavity is a pathway for numerous neurovascular structures and musculature. In terms of vasculature, several vessels traverse this space including the maxillary artery and its associated branches (the deep temporal artery, middle meningeal artery, inferior alveolar artery, and buccal artery), pterygoid venous plexus, maxillary vein, and middle meningeal vein [2]. In terms of nerves, the trigeminal nerve’s mandibular and sensory branches, the facial nerve’s chorda tympani branch, and the otic ganglion all contribute to motor, sensory, and autonomic function to the surrounding area. Finally, within the fossa, the muscles for mastication and swallowing include the medial and lateral pterygoid, tensor veli palatini, and levator veli palatini [2].

Neoplasms and infections are the most common pathologies affecting the ITF. Infections of the ITF are rare, but when they do occur, they are thought to arise from contiguous transmission of bacteria from the oral cavity through the masseteric fascia [3]. Tooth extractions and odontogenic infections, which use the aforementioned method for access, are the most common inciting events. Based on a systematic review of IFA, there have been 67 documented cases published in the last century [4]. Here we present a patient with IFA after wisdom teeth extraction requiring multidisciplinary medical management to achieve adequate source control. We hypothesize this patient’s unique clinical course to be a consequence of coaggregation between *Streptococcus anginosus* and *Eikenella corrodens*, as well as delayed diagnosis.

## Case report

**Fig. 1.**
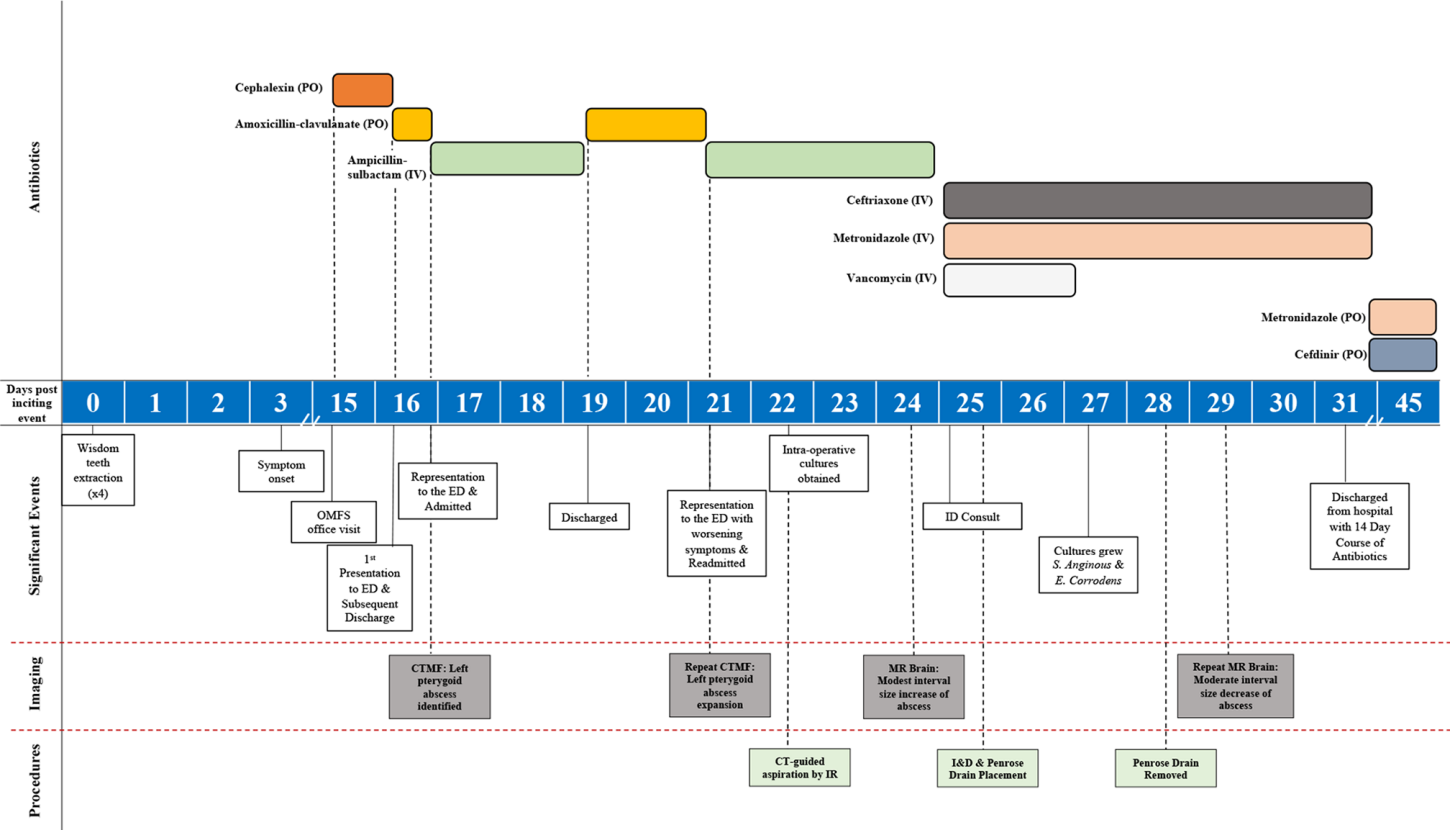
Case report timeline.

A previously healthy 20-year-old male presented to the emergency department (ED) for the second time in the preceding 24 h complaining of left-sided face pain and increased difficulty opening his mouth. His medical history revealed that he had four wisdom teeth extracted 2 weeks prior without any acute complications (Fig. 1). Several days following the procedure, his pain progressively worsened. To address the pain, he consulted his oral and maxillofacial surgeon (OMFS). Clinical evaluation revealed an acute onset of unilateral parotid gland tenderness accompanied by trismus, prompting his OMFS to prescribe Cephalexin (first-generation cephalosporin antibiotic) as initial treatment for suspected bacterial sialadenitis. When he arrived at the ED, he was given oxycodone (opiate agonist) and acetaminophen (non-opioid analgesic) which provided adequate pain relief. There was no imaging or laboratory testing done at the time. In response to inadequate clinical improvement, medical intervention was modified to amoxicillin-clavulanate (a penicillin/beta-lactamase inhibitor antibiotic) to better cover for oral anaerobes. Concurrently, analgesic management was initiated utilizing morphine (opiate agonist) along with non-pharmacological sialagogues. He was discharged home with prompt OMFS follow-up the next day. That same night he subsequently developed 10/10 left-sided jaw pain that resulted in his current presentation to the ED.

**Fig. 2.**
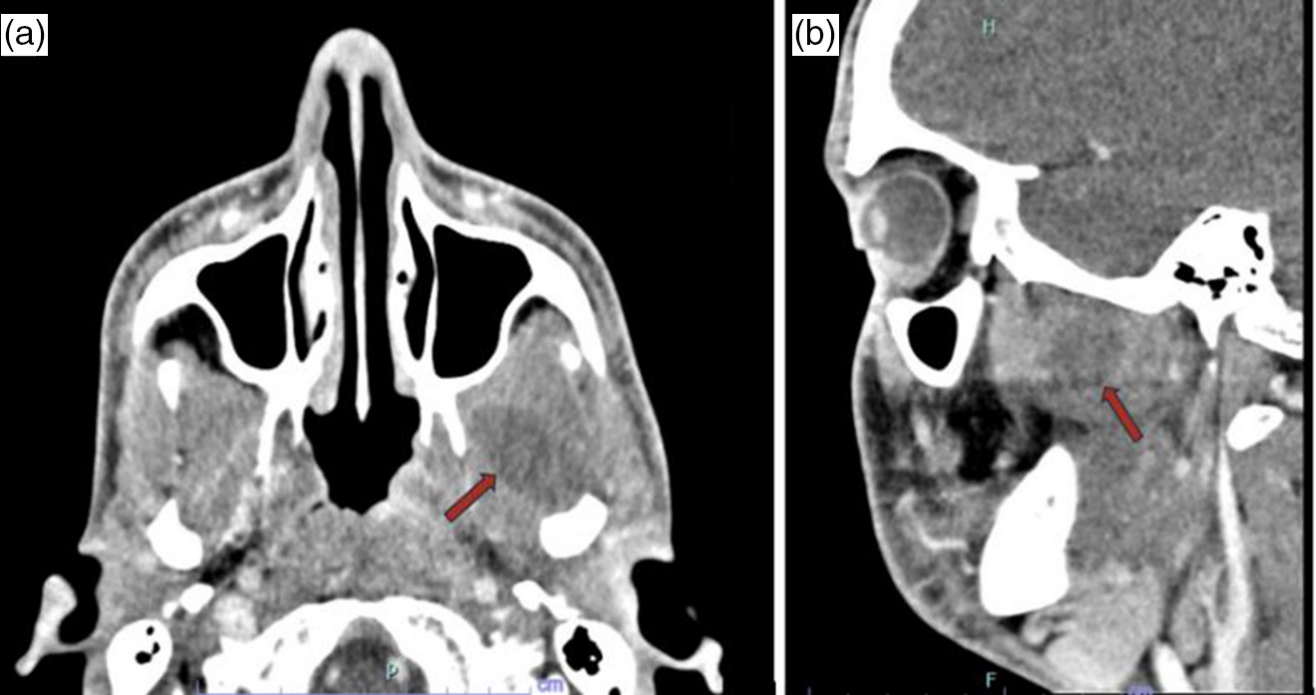
Rim-enhancing lesion in the left pterygoid musculature. (**a**) Axial and sagittal (**b**) view of maxillofacial CT with contrast demonstrating a left pterygoid musculature abscess measuring 2.7×1.6×1.6 cm with surrounding oedema in the medial and lateral pterygoids.

On presentation, he had a fever of 38.5 °C and was tachycardic to 139 beats per minute (bpm) but was otherwise normotensive with a normal respiratory rate and oxygen saturation. Physical examination revealed a diaphoretic young man who was writhing in pain. He had trismus as a result of pain and oedema around his left jaw and face. This area was extremely tender to palpation. No leukocytosis was demonstrated on complete blood count (CBC). The complete metabolic panel (CMP) revealed no electrolyte or liver enzyme derangements. Maxillofacial computed tomography (CTMF) demonstrated a rim-enhancing lesion in the left pterygoid musculature, with surrounding oedema in the medial and lateral pterygoids, consistent with a pterygoid abscess (Fig. 2a, b). He was transitioned to intravenous (IV) ampicillin-sulbactam and admitted to the hospital. Ear, nose, and throat (ENT) physicians were consulted, and they determined the abscess was rather difficult to access and recommended 24 h of IV antibiotics before reevaluating for surgical interventions. Upon reexamination, he was discharged in the early morning with an outpatient OMFS appointment set for the same day, owing to his clinical improvement and well-controlled pain with analgesics.

**Fig. 3.**
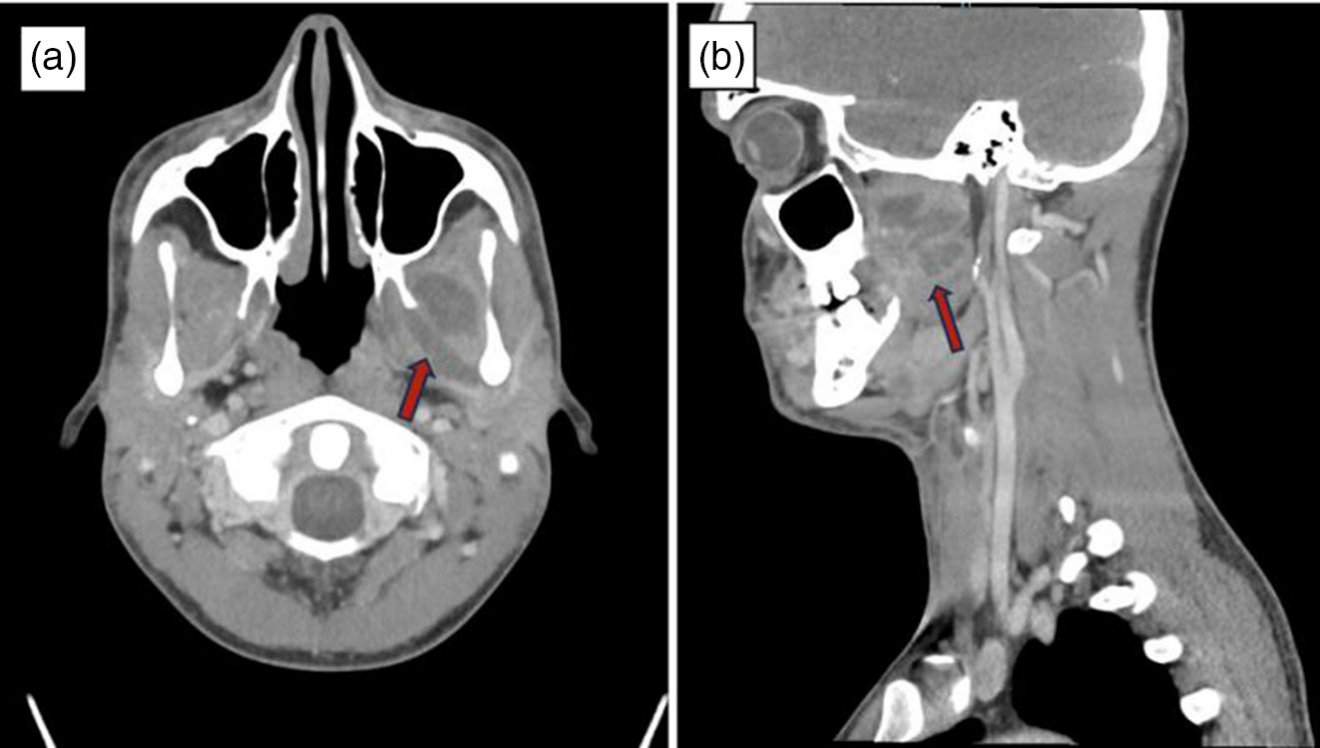
Interval abscess enlargement. Interval enlargement of left-sided masticator space fluid collection with multiple loculations, now with more inferior spread centered about the left lateral pterygoid musculature, with a new lateral pterygoid muscle component (**a**) measuring 3.1×0.6×0.9 cm, and a new medial pterygoid muscle component (**b**) measuring 0.9×2.3×0.8 cm.

He returned to the ED 2 days later with uncontrollable pain, severe swelling, and new odynophagia, left-sided neck pain, and voice muffling. Vitals were significant for hypothermia to 36.0 °C, normotension, and tachycardia to 118 bpm with normal respiratory rate and oxygen saturation. CBC demonstrated a leukocytosis of 15.4 K µl^−1^ (normal 4.0–11.0) with a left shift. CMP was within the acceptable range. C-reactive protein was elevated at 192.8 mg l^−1^ (normal ≤4.9) and the erythrocyte sedimentation rate was elevated at 39 mm hr^−1^ (normal 0–15). He met sepsis criteria and was readmitted to the hospital for IV antibiotics. Repeat CTMF revealed an expansion of the known abscess as well as new medial and lateral pterygoid muscle involvement (Fig. 3a, b). Due to previous improvement while on IV ampicillin-sulbactam, this antibiotic was restarted in favour of amoxicillin-clavulanate. Several specialties, including neurosurgery, interventional radiology (IR), and ENT, were consulted at this time. Given the anatomic intricacy of the abscess location, neurosurgery recommended IR intervention. The following day, IR performed a CT-guided aspiration of the abscess in the left masticator area and acquired intra-procedural cultures.

Two days post-aspiration, magnetic resonance imaging (MRI) of the brain, orbit, face, and neck with and without contrast demonstrated a modest interval size growth of the abscess with new enhancement propagating through the temporalis muscle in a cephalad direction. The next day, infectious diseases was consulted, and it was recommended that ampicillin-sulbactam be discontinued. In its place, vancomycin (glycopeptide antibiotic) was started for methicillin-resistant *Staphylococcus aureus* coverage, along with ceftriaxone (third generation cephalosporin antibotic) and metronidazole (nitroimidazole antibiotic) to maintain coverage for Gram-positive/negative organisms and anaerobes, respectively. The patient was operated on the same day by an ENT specialist, who performed a successful incision and drainage (I&D) and Penrose drain placement.

Intra-operative cultures later grew *S. anginosus* and *E. corrodens* which were susceptible to ceftriaxone, penicillin, and vancomycin; blood cultures remained negative throughout his admissions. A moderate interval decrease in the lateral and medial pterygoid muscle abscess was seen on repeated MR brain/orbit of the face and neck. Outpatient parenteral antimicrobial therapy (OPAT) with ceftriaxone for 1 week was recommended by infectious diseases. However, because he was starting school and moving into a new house, he asked for per os (PO) antibiotics over OPAT. A compromise was reached so the patient could be discharged on PO cefdinir (third generation cephalosporin) as an alternative for ceftriaxone due to its similar coverage, metronidazole, and chlorhexidine gluconate (antimicrobial oral rinse) mouthwash for 2 weeks with stringent and frequent OMFS monitoring.

Although he completed his antibiotics and the infection completely resolved, he later developed long-term consequences of severe pain in the left jaw from scar tissue formation, which necessitated surgical debridement by OMFS. A year after his final inpatient discharge, aside from experiencing some residual pain, the patient was found to be well with no repeated episodes of ITF infection.

## Discussion

Formerly known as *Bacteroides corrodens*, *Eikinella corrodens* is a genus of the Neisseriaceae family of bacteria and a facultative anaerobic Gram-negative bacillus [5, 6]. *Eikinella* is endogenous to mucosal surfaces and is most prominent in the oropharynx and upper respiratory tract microbiota. The majority of infections occur in the head and neck with a propensity to affect both adults and children [7]. According to a recent systematic review of IFA in adults, two cases of *Bacteroides* species have been identified as the etiological organism for infratemporal abscesses [4]. In a literature review of 58 cases involving *E. corrodens* head and neck infections, only one case involved the infratemporal fossa, highlighting its rarity [8]. The patient in the aforementioned case was a 7-year-old child who developed a medial pterygoid abscess secondary to parapharyngeal space lymphadenitis abscess [9]. Brain, thyroid, orbital/periorbital, tonsil/peritonsillar abscesses, and sinusitis accounted for the vast majority of *E. corrodens* infections.

Three unique streptococcal species, including *S. anginosus*, *S. intermedius*, and *S. constellatus*, make up the *Streptococcus anginosus* group (SAG), also known as the *Streptococcus milleri* group [10]. *S. anginosus* species are anaerobic facultative Gram-positive cocci (AFGPC) that are catalase-negative. SAG commonly colonizes healthy human flora, including the genitourinary (GU), gastrointestinal (GI), and respiratory tracts, without causing any harm to the host. Tissue trauma and underlying comorbidities such as malignant tumours or diabetes can lead to invasive disease, most commonly in the form of an abscess [11]. Clinical studies have shown certain SAG species have a tendency to target particular tissues, with *S. anginosus* causing invasive disease of the GU and GI tract, *S. constellatus* causing infections of the skin, respiratory tract, and soft tissues, and *S. intermedius* causing infections of the head and neck [12–14].

Bacterial coaggregation is the process by which distinct microbial species adhere together to form a multi-species biofilm that aids in host immune system evasion [15]. In experimental models, *E. corrodens* was found to have weak virulence on its own but exhibited increased pathogenicity when co-existing with other SAG organisms, most notably with *S. anginosus* and *S. constellatus* [16]. Despite the frequent coaggregation of *S. anginosus* and *E. corrodens*, no growth stimulation was observed. This contrasts with instances where *E. corrodens* did demonstrate growth stimulation with other streptococcal species. Encapsulation, however, is a different pathogenic component that may increase virulence and encourage the development of abscesses. In one study, 50 % of mice developed an abscess after being inoculated with AFGPC. Mice that did not develop an abscess were then inoculated with capsule-positive *Bacteroides* species or other capsular material which resulted in abscess development in 73 % of the mice. The subsequently cultivated strains were discovered to be highly capsulated and easily produced abscesses upon reinoculation into mice, indicating enhanced pathogenicity and synergy between these bacteria [17].

A systematic review demonstrated that *E. corrodens* co-infection with another organism was present in 74.1 % of paediatric head and neck infections; the most common co-infection was with Viridians group streptococcus, followed by *Staphylococcus aureus* [8]. Two of the 58 patients had *S. anginosus* and *E. corrodens* co-infections; one resulted in a thyroid abscess and the other in suppurative thyroiditis. Although co-infection of *E. corrodens* with specific *Streptococcus* and *Staphylococcus* has been published, we provide a new case to add to the literature. To the best of our knowledge, our patient is the first case of *E. corrodens* and *S. anginosus* co-infection as the cause of an IFA that has been described. Although the microorganisms isolated from the ITF were not identified in our patient’s oral cavity, serving as one of the limitations for our conclusions, *E. corrodens* and *S. anginosus* are endogenous to mucosal surfaces, serving as a plausible mechanism that dental manipulation was the inciting event that allowed for contiguous transmission of bacteria from the oral cavity through the masseteric fascia and into the infratemporal space. There were no other presumable sources of infection given that blood cultures were negative, and no skin lesions were identified. We hypothesize that the protracted and challenging clinical course of our patient was partially brought on by the increased virulence of these bacteria in combination as opposed to isolation.

Although rare, this patient’s presentation emphasizes the need to include infratemporal abscesses in the differential diagnosis, especially in the context of recent dental treatment, as the implications can be catastrophic if not treated swiftly. In order to accomplish source control, timely diagnosis is also required. From the time of symptom onset, it took 12 days to be seen by medical personnel, and on day 13 the abscess was identified during his second ED visit that day. This could have been expedited by closer outpatient follow-up for symptom monitoring and a lower threshold for initiating antibiotics. In addition, a lower threshold for imaging should be considered when protracted symptoms are apparent. With this patient’s clinical picture in mind, it introduces the concept that whenever an abscess involving *Streptococcus* is encountered in a clinical setting, a strong suspicion for *Eikenella* co-infection is required, especially because it has fastidious growth requirements and may fail to speciate. This may serve a clinically meaningful purpose when considering antibiotic de-escalation as empiric coverage may still be required.

## Conclusion

To the best of our knowledge, we report the first case of an infratemporal abscess caused by *Eikenella corrodens* and *Streptococcus anginosus* co-infection. A delay in diagnosis can result in prolonged clinical courses and life-threatening complications. A multidisciplinary approach including multiple surgical and medical subspecialists is frequently required to deliver a prompt diagnosis and optimize patient outcomes.
